# Correspondence

**Published:** 1872-04

**Authors:** H. B. Boswell

**Affiliations:** Dentist


BRISTOL, INDIANA,

February 13th, 1872.

DR. TAFT, *Dear Sir:* The following is a case of hemorrhage, resulting from the extraction of a tooth, that recently came under my observation, which defied all the usual remedies for its arrest.

I noticed an article in the April number of the *Medical Record* (New York) taken from the DENTAL REGISTER, to the effect that Dr. L. N. Hutchinson, of Big Rapids, Michigan had a similar case which he arrested by the use of Plumb's Acetas, and I have had occasion to test its merits and with the following result:

February 10th, 1872, Mr. C——, aged fifty-five years, of a hemorrhagic diathesis, applied to Dr. A——, a physician, at 9 o'clock, A. M., for the removal of the left inferior central incisor, which was accomplished, and the patient returned home. At 1 o'clock, P. M., a severe hemorrhage commenced which resisted all the domestic applications of which he was in possession; becoming alarmed at the continued flow of blood, he applied to another physician who filled the socket with pellets of cotton saturated with *Ferri per. Sulph.*, and the patient again returned home. But the hemorrhage still continued, a constant flow of blood, issuing from the socket. At 7 o'clock, P. M., the patient becoming weak and nervous, again applied to Dr. A——, who applied more *Ferri per. Sulph.*, both dry and liquid, also tannin, without the least effect. At this point I happened into Dr. A——'s office, and seeing the state of affairs, suggested a trial of Plumb's Acetas, which he seconded, and requested me to make the application, which I did in the following manner: Mixed Plumb's Acetas two parts with tannin one part and rubbed fine with a spatula. I then removed all the cotton from the socket and cleansed the mouth thoroughly, then introduced a very small pellet of cotton which had been previously rolled in the preparation into the socket and forced it down with a probe, then forced in another, a little larger, in the same manner, also a third, which filled the socket; then made a compress by rolling a strip of cotton cloth three-quarters of an inch wide into a flat roll and placed edgewise between the teeth on either side so that when the mouth was closed the upper teeth forced it firmly down upon the gum. The hemorrhage immediately ceased and the patient had no further trouble. I am satisfied the Plumb's Acetas did the business and I now keep it in my cabinet for further trials.

H. B. Boswell, Dentist.

